# Machine Learning Methods for Hypercholesterolemia Long-Term Risk Prediction

**DOI:** 10.3390/s22145365

**Published:** 2022-07-18

**Authors:** Elias Dritsas, Maria Trigka

**Affiliations:** Department of Computer Engineering and Informatics, University of Patras, 26504 Patras, Greece; trigka@ceid.upatras.gr

**Keywords:** cholesterol, hypercholesterolemia, long-term prediction, machine learning, data analysis

## Abstract

Cholesterol is a waxy substance found in blood lipids. Its role in the human body is helpful in the process of producing new cells as long as it is at a healthy level. When cholesterol exceeds the permissible limits, it works the opposite, causing serious heart health problems. When a person has high cholesterol (hypercholesterolemia), the blood vessels are blocked by fats, and thus, circulation through the arteries becomes difficult. The heart does not receive the oxygen it needs, and the risk of heart attack increases. Nowadays, machine learning (ML) has gained special interest from physicians, medical centers and healthcare providers due to its key capabilities in health-related issues, such as risk prediction, prognosis, treatment and management of various conditions. In this article, a supervised ML methodology is outlined whose main objective is to create risk prediction tools with high efficiency for hypercholesterolemia occurrence. Specifically, a data understanding analysis is conducted to explore the features association and importance to hypercholesterolemia. These factors are utilized to train and test several ML models to find the most efficient for our purpose. For the evaluation of the ML models, precision, recall, accuracy, F-measure, and AUC metrics have been taken into consideration. The derived results highlighted Soft Voting with Rotation and Random Forest trees as base models, which achieved better performance in comparison to the other models with an AUC of 94.5%, precision of 92%, recall of 91.8%, F-measure of 91.7% and an accuracy equal to 91.75%.

## 1. Introduction

Cholesterol is a form of fat and a key component of cells. It plays a very important role in health as it participates in the synthesis of hormones, in the production of vitamin D and in the digestion and assimilation of fats. The molecules that result from the binding of cholesterol to proteins are called lipoproteins and are categorized into “bad” LDL cholesterol and “good” HDL cholesterol. LDL cholesterol is responsible for transporting cholesterol molecules from the liver to tissues and organs, while HDL cholesterol transports cholesterol molecules from tissues back to the liver [1,2].

Cholesterol is calculated in milligrams (mg) of cholesterol per deciliter (dL) of blood. An effect below 200 mg/dL (5.2 mmol/L) is ideal. A level somewhere between 200 and 239 mg/dL (5.2–6.2 mmol/L) is marginally below the high-risk number. A value above 240 mg/dL (6.3 mmol/L) is the high-risk limit. Total cholesterol results from the sum of HDL and LDL values [3].

In HDL, a value below 40 mg/dL (1 mmol/L) for men and 50 mg/dL (1.3 mmol /L) for women is low. This increases the risk of cardiovascular problems. A normal HDL cholesterol level is between 40 and 49 mg/dL (1–1.3 mmol/L) for men. For women, it is between 50 and 59 mg/dL (1.3–1.5 mmol/L). When this level is higher than 60 mg/dL (1.6 mmol/L), there is increased defense against coronary heart disease [4].

In LDL cholesterol, a value below 100 mg/dL (2.6 mmol/L) is ideal. The value between 100 and 129 mg/dL (2.6 and 3.3 mmol/L) is close to ideal, while between 130 and 159 mg/dL (3.4 and 4.1 mmol/L) it is marginally increased. LDL between 160 and 189 mg/dL (4.1 and 4.9 mmol/L) is considered high. When it exceeds 190 mg/dL (4.9 mmol/L) it is very high. It is recommended that LDL cholesterol be below 70 mg/dL (1.8 mmol/L) [4].

Although cholesterol is essential for the human body, high levels in the blood are associated with vascular damage and cardiovascular disease. Sometimes, our body produces more cholesterol than it needs, and this excess circulates in the blood. High blood cholesterol levels can cause blood vessels to clot and increase the risk of atherosclerotic plaque, coronary heart disease, angina, heart attack, peripheral arterial disease and stroke [5,6,7].

It should be noted that a high level of cholesterol is estimated to cause 2.6 million deaths (4.5% of total) and 29.7 million disability-adjusted life years (DALYS), or 2% of total DALYS. Raised total cholesterol is a major cause of disease burden in both the developed and developing world [8].

High cholesterol levels (hypercholesterolemia) may be due to lifestyle, genes (heredity) or, secondarily, to health conditions such as kidney disease. Factors responsible for the increase in cholesterol are poor diet, lack of physical activity, smoking, certain medications as well as pathological conditions such as diabetes, obesity, chronic kidney disease, HIV, hypothyroidism and polycystic ovary syndrome [9,10].

Diet plays an important role in improving cholesterol levels. High blood cholesterol levels are significantly reduced with lower consumption of fatty foods (meat, dairy, cold cuts), increased fibre intake (fruits, legumes) and frequent consumption of fatty fish rich in omega-3 fatty acids (sardines, mackerel). In addition, daily physical exercise will have beneficial effects in lowering cholesterol [11].

Information and communication technologies (ICTs), and especially the fields of artificial intelligence (AI) and machine learning (ML), are moving in this direction. ML techniques now play an important role in the early diagnosis of various diseases, such as diabetes (as classification [12] or regression task for continuous glucose prediction [13,14]), hypertension [15], COPD [16], COVID-19 [17], CVDs [18], stroke [19], CKD [20], ALF [21], hepatitis [22], sleep disorders [23], cancer [24], etc.

Especially, in this study, ML models are explored to estimate the long-term risk of hypercholesterolemia occurrence with the aid of various risk factors. The main contribution of this article is a comparative evaluation of several supervised learning classifiers to find the one with the highest sensitivity and separability, which means that it is the most appropriate to correctly identify those at high risk. An essential aspect of the data mining process is data pre-processing in the context of which data cleaning, features selection and class balancing were applied. Several performance metrics are utilized to evaluate the classifiers’ performance, such as precision, recall, F-measure, accuracy and AUC. Performance analysis showed that data quality has a significant impact on the training of efficient models. Finally, the quantitative analysis demonstrated that the soft voting is the most competent model, and thus it constitutes the main suggestion of this study.

The rest of the paper is organized as follows. Section 2 describes the relevant works with the subject under consideration. In Section 3, a dataset description and analysis of the methodology followed is made. In addition, in Section 4, we discuss the acquired research results. Finally, conclusions and future directions are outlined in Section 5.

## 2. Related Work

The estimation of individual risk for the development of a chronic condition has gained high popularity in the medical field. Therefore, in predictive analytics and, especially machine learning, numerous studies have been conducted to estimate personal risk using various data related to socioeconomic features (age group, gender and race), behavioural data and, recently, clinical risk factors. In this direction, we will present some recent studies’ outcomes that use machine learning techniques to predict hypercholesterolemia.

Familial hypercholesterolemia (FH) is a dominant genetic condition with an increased risk of coronary artery disease (in untreated cases) [25]. Machine learning-based strategies can lead to the effective identification of high-risk patients to enhance FH management.

First, the authors in [26] selected three machine learning algorithms including a classification tree, a gradient boosting machine and a neural network to predict the presence of FH in two different cohorts. The evaluation was based on the area under the ROC curve. The findings have shown the superiority of machine learning models against the clinical Dutch Lipid Score in predicting carriers of FH-causative mutations.

In [27], a Random Forest classifier was developed to identify potential FH patients using electronic health record (EHR) data. The model was trained on 197 known patients and 6590 without FH, achieving a positive predictive value (PPV) of 0.88 and a sensitivity of 0.75 on a hold-out test set. The accuracy of the classifier’s predictions was further evaluated by a chart review of patients at risk of FH not included in the original dataset. The classifier correctly categorized 84% of patients at the highest probability threshold. Finally, the same classifier was validated on an external dataset from the Geisinger Healthcare System and achieved a PPV of 0.85.

Moreover, in [28], the authors developed a model for predicting hypercholesterolemia using a comprehensive set of body fat mass variables based on machine learning techniques, in addition to studying the correlation between body fat mass and hypercholesterolemia. They obtained the area under the receiver operating characteristic curve value of 0.739 and the Matthews correlation coefficient value of 0.36 in the model using the correlation-based feature subset selection and the Naive Bayes algorithm.

A machine learning approach for the prediction of cholesterol levels via regression using non-invasive and easy-to-collect data (clinical and anthropometric) is presented in [29]. In addition, clustering analysis is carried out to identify different groups of patients sharing some characteristics and give valuable information to clinical experts for diagnosis or prognosis.

Moreover, the authors in [30] aimed to compare the performance of various machine learning models to predict the prevalence of hypercholesterolemia associated with exposure to lead, mercury and cadmium. Five machine learning models, such as Logistic Regression, K-Nearest Neighbors, Decision Trees, Random Forest, and Support Vector Machines were constructed, and their predictive performance were compared. Finally, the Support Vector Machine model was the most accurate, and the logistic regression model had the highest area under the ROC curve of 0.718 (95% CI: 0.688–0.748).

In [31], the authors developed a high accuracy (97.45%) convolutional neural network-based Android application that determines cholesterol levels in a person’s body by capturing the image of the iris. A user with high cholesterol levels has a white–greyish circle on the outer circle of the iris.

Finally, the authors in [32] constructed a dataset based on the ELSA database, aiming at the prognosis of high cholesterol (hypercholesterolemia), targeting the elderly office workers. Naive Bayes, Support Vector Machines, Artificial Neural Network using two hidden layers, 5-Nearest Neighbors, Rotation Forest, Decision Trees, Logistic Model Trees and Random Forest were applied on the constructed dataset using a 10-fold cross-validation experimentation setup. The best overall performance was obtained with the Logistic Model Trees model, which performed best both concerning accuracy and recall metrics.

## 3. Materials and Methods

This section describes the dataset under consideration and the methodology adopted for determining the risk of being diagnosed with hypercholesterolemia.

### 3.1. Dataset Description

Our experimental results were based on the dataset of research work [32], which is derived from the English Longitudinal Study of Aging (ELSA) [33]. The initial features set included 106 variables, with 61 being nominal and 45 numerical attributes, and all the participants were over 50 years old. From the features list, we excluded socioeconomic data, including the type of employment, education, income, residence type, marital status, the number of children and insurance type. Moreover, after data cleaning and feature selection, the final list of features was reduced to 13, the number of participants to 350, and all the attributes (13 as input to ML models and 1 for target class) are described as follows:

- **Age** (years) [34]: This feature refers to the age of a person who is over 50 years old. It is numerical data.
- **Gender** [34]: This feature refers to a person’s gender. The number of men is 172 (49.15%), while the number of women is 178 (50.85%) It is nominal data.
- **BMI** (Kg/m^2^) [35]: This feature captures the participant’s body mass index. It is numerical data.
- **Waist** (cm) [36]: It is the measurement taken around the abdomen at the level of the umbilicus. It is numerical data.
- **SBP** (mmHg) [37]: This feature captures the participant’s systolic blood pressure. It is numerical data.
- **DBP** (mmHg) [37]: This feature captures the participant’s diastolic blood pressure. It is numerical data.
- **Hypertension** [38]: This feature refers to whether a participant is hypertensive or not. The percentage of participants who have hypertension is 58.9%. It is nominal data.
- **HDL** (mg/dL) [2]: This feature captures the participant’s high-density lipoprotein. It is numerical data.
- **LDL** (mg/dL) [2]: This feature captures the participant’s low-density lipoprotein. It is numerical data.
- **TotChol** (mg/dL) [2]: This feature captures the participant’s total cholesterol. It is numerical data.
- **Physical Activity** [39]: This feature captures the participant’s physical activity and has 4 categories (high 2.6%, medium 11.2%, low 55.4% and very low 30.8%). It is nominal data.
- **Alcohol Consumption** [40]: This feature refers to whether this participant consumes alcohol or not. The percentage of participants who consume alcohol more than normal is 44.1%. It is nominal data.
- **Diabetes** [41]: This feature refers to whether the participant has been diagnosed with diabetes or not. The percentage of participants who suffer from diabetes is 20.6%. It is nominal data.
- **Hypercholesterolemia**: This feature stands for whether the participant has been diagnosed with hypercholesterolemia. The percentage of participants who have been diagnosed with hypercholesterolemia is 44.6%. It is nominal data.

### 3.2. Hypercholesterolemia Risk Prediction

Supervised machine learning models have become an important asset for clinicians and healthcare providers as they allow them to evaluate the long-term risk of a condition occurrence based on several risk factors. More specifically, here, our purpose is to formulate a binary classification problem with target class c = “HyperChol” (hypercholesterolemia occurrence) or c = “Non-HyperChol” (non-occurrence of the hypercholesterolemia) and design models which will achieve high recall and area under curve (AUC) to ensure that instances with hypercholesterolemia can be accurately classified.

Assuming an instance with an unknown class label (HyperChol, Non-HyperChol), the trained ML models will predict its class based on the features’ values and thus the risk of occurring hypercholesterolemia in the long term.

The proposed methodology includes some specific steps, namely, data preprocessing, feature ranking, classification models training and performance evaluation.

#### 3.2.1. Data Preprocessing

Data quality is a prerequisite for the development of efficient models suitable for the correct identification of healthy and with hypercholesterolemia instances. Hence, to ensure data validity, several preprocessing steps are usually applied. Data quality is ensured via the application of data cleaning methods, selecting the most appropriate for the data under consideration, such as excluding unnecessary or duplicate values, avoiding typos, handling missing values, data imputation, etc. [42]. As for the current dataset, we selected to remove instances whose feature values are missing and not valid (namely, out of the normal limits).

Moreover, the skewed class distribution constitutes a factor that can create ML models of poor performance. For this purpose, the imbalanced distribution of participants among the HyperChol and Non-HyperChol classes was tackled by employing SMOTE [43]. SMOTE uses a 5-NN classifier to create synthetic data on a minority class, i.e., HyperChol, which is oversampled such that the instances in two classes are equally distributed (i.e., 50%–50%). In Table 1, we present the minimum, maximum, mean and standard deviation of the numerical features in the balanced data.

#### 3.2.2. Features Ranking

**Figure 1:**
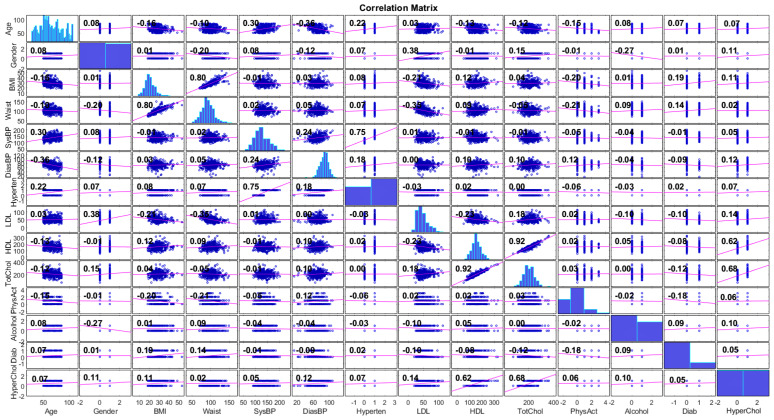
Pearson correlation analysis.

We employed four ranking methods to understand the importance of a feature in the target class. First, we applied the Pearson correlation coefficient [44] to evaluate the strength of association between all features and especially the worth of a feature in predicting the hypercholesterolemia class. Figure 1 demonstrates the outcomes of the correlation analysis. In the correlation matrix, we observe that the highest linear correlation of 0.92 is captured between total cholesterol levels and HDL, and total cholesterol levels and LDL. The next but still high linear relationship of rank 0.80 is noted between waist and BMI features, a high association of 0.75 is shown between hypertension and systolic blood pressure, while hypertension has a low association of 0.18 with the diastolic blood pressure. A moderate positive association of 0.3 is shown between age and systolic blood pressure. However, diastolic blood pressure has a moderate negative relationship with age. Finally, the target class records a moderate association of 0.68 and 0.62 with the total cholesterol and HDL, respectively, while a low relationship seems to exist with the other features.

Then, we applied the Information Gain method (InfoGain) [45] which evaluates the worth of a feature by measuring the information gain with respect to the class, according to the formula

(1) $$InfoGain\left(c,y_{ij}\right)=H\left(c\right)-H\left(cy_{ij}\right),j\in 1,2,\ldots ,n$$

where H(c) and $H(cy_{ij})$ are the entropy of the hypercholesterolemia class and the conditional entropy of the class given the feature *j* $y_{ij}$ of an instance *i*. From (1), we see that this measure captures the difference in entropy before and after the split of a feature set based on a specific $y_{ij}$. Alternatively, it shows the uncertainty reduction after splitting the set on a feature. The best feature for splitting is the one with the highest information gain.

Furthermore, we employed the Gain Ratio (GR) method [46] which is calculated as $GR\left(y_{ij}\right)=\frac{H\left(c\right)-H\left(cy_{ij}\right)}{H(y_{ij})}$, where $H\left(y_{ij}\right)=-p_{y_{ij}}log_{2}\left(p_{y_{ij}}\right)$ is the entropy of an instance with feature $y_{ij}$ (with $p_{y_{ij}}$ denoting the probability of selecting feature $y_{ij}$), $H\left(c\right)=-p_{c}log_{2}\left(p_{c}\right)$ is the entropy of class *c* (with $p_{c}$ be the probability of selecting an instance in class *c*) and $H(cy_{ij})$ being the conditional entropy of feature $y_{ij}$ given class *c*. Gain ratio indicates the relevance of a feature and selects the ones that maximize gain ratio based on the probability of each feature value.

Finally, the Random Forest classifier was selected to measure the importance of the features. Random Forest creates a forest of trees and per tree measures the discrimination ability of a potential feature to create the optimal split, namely the one that separates the instances of the two classes, using the Gini impurity.

In Table 2, we summarize features’ importance in the balanced dataset concerning the hypercholesterolemia class. All considered methods show that TotChol and HDL features are of the highest importance for the prediction of hypercholesterolemia. In addition, we observe that DiasBP is the next most important for the three out of four methods. Moreover, InfoGain and Gain ratio, due to their relationship, assign the features in the same order except for physical activity and hypertension. Since all features are among the risk factors that are utilized by the clinicians for the diagnosis and management of hypercholesterolemia, the models’ training and validation will be based on all of them. Finally, given that ML models can be retrained with more data, their importance will be re-investigated.

### 3.3. Data Exploration

In this subsection, after data preprocessing and features ranking, we will analyze the association between features and the HyperChol class.

**Figure 2:**
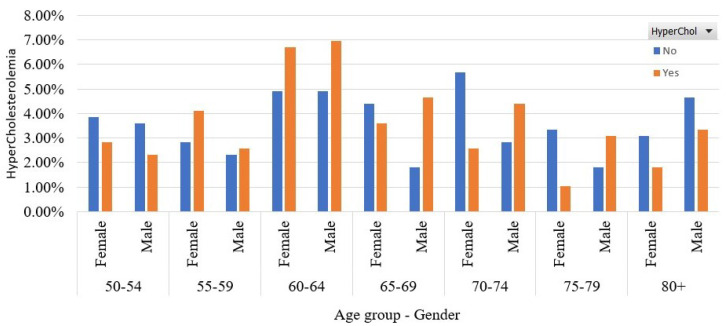
Participants’ distribution per age group and gender type in the balanced dataset.

Initially, in Figure 2, we present the participants’ distribution according to their age group and their gender. Most of them who have HyperChol belong to the age group 60–64, where men’s and women’s percentages are approximately similar. In addition, from this figure, we observe that HyperChol prevails in women who are between 50 and 59 and men who are older than 65 years old.

**Figure 3:**
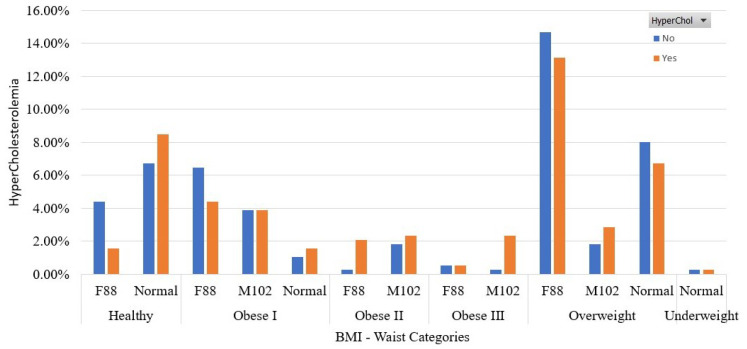
Participants’ distribution in terms of BMI and waist categories in the balanced dataset.

In Figure 3, we present the participants’ distribution in terms of the BMI and waist categories. To define the BMI categories, we used the following rules [13]:

(1) BMI<18.5: underweight
(2) 18.5≤BMI<25: healthy
(3) 25≤BMI<30: overweight
(4) BMI≥30.0: obesity
  (a) Class I: 30≤BMI<35
  (b) Class II: 35≤BMI<40
  (c) Class III: BMI≥40 (severe obesity).

Cut-off points for the waist size were considered 88 cm for women and 102 cm for men [47]. Based on these points, the labels F88 and M102 capture women and men with waist circumference higher than 88 and 102 cm, respectively. The label “normal” refers to men and women with waist sizes lower than 88 and 102 cm, correspondingly. From this figure, we see that most of the participants with HyperChol are distributed in healthy and overweight and obese I categories of BMI. Moreover, when HyperChol and overweight classes coexist, the women with waists higher than the cut-off point are much more than men. In addition, some instances have HyperChol and are overweight, but they have normal waist size.

**Figure 4:**
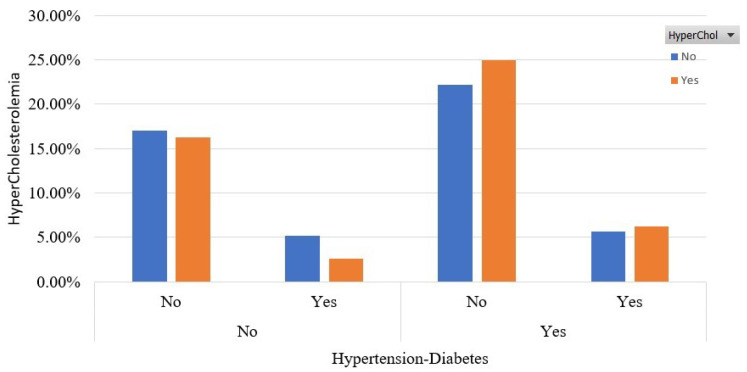
Participants’ distribution for both diabetes and hypertension in the balanced dataset.

Furthermore, in Figure 4, we capture the coexistence of hypertension and diabetes diseases in relation to HyperChol. We see that 25% of the participants have HyperChol, are hypertensive and have not been diagnosed with diabetes. A small percentage of 6% fulfills all criteria; 16% of the participants who have HyperChol do not suffer from hypertension and diabetes.

**Figure 5:**
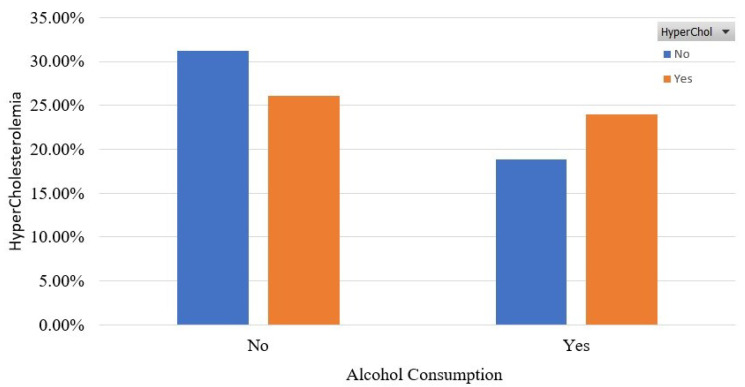
Participants’ distribution in terms of alcohol consumption in the balanced dataset.

**Figure 6:**
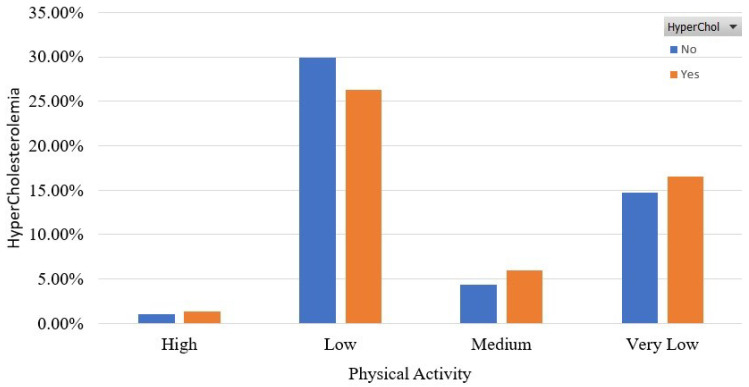
Participants’ distribution in terms of physical activity in the balanced dataset.

Figure 5 and Figure 6 show the association of HyperChol with participants’ habits in terms of alcohol consumption and physical activity. It is shown that the HyperChol participants are roughly the same distributed among the two possible values of the alcohol consumption feature. Finally, as for Figure 6, it should be noted that most of the HyperChol participants are very low or low physically active.

### 3.4. Machine Learning Models

Several models have been selected to evaluate their prediction performance. More specifically, Naive Bayes, Support Vector Machine, Artificial Neural Network, K-NN, Rotation Forests, Decision Trees, Logistic Model Trees, Random Forest, and Ensemble Learning (Stacking and Soft Voting) classification methods will be presented. We assume that each instance *i* in the dataset is represented by a features vector $y_{i}={\left[\begin{array}{c} y_{i1},y_{i2},y_{i3},\ldots ,y_{in} \end{array}\right]}^{T}$, where *n* is the number of the features.

#### 3.4.1. Naive Bayes

Naive Bayes(NB) classifies an instance $y_{i}$ at that class *c* for which $P\left(c\right|y_{i1},\ldots ,y_{in})$ is maximized (under the assumption that the features are highly independent [48]). The conditional probability is defined as $P\left(c\right|y_{i1},\ldots ,y_{in})=\frac{P\left(y_{i1},\ldots ,y_{in}|c\right)P\left(c\right)}{P\left(y_{i1},\ldots ,y_{in}\right)}$, where $P\left(y_{i1},\ldots ,y_{in}|c\right)=\prod_{j=1}^{n}P\left(y_{ij}|c\right)$ is the features probability given class and $P\left(y_{i1},\ldots ,y_{in}\right),P\left(c\right)$ are the prior probability of features and class, respectively. The estimated class is derived by maximizing $P\left(c\right)\prod_{j=1}^{n}P\left(y_{ij}|c\right)$, where c∈{HyperChol,Non−HyperChol}.

#### 3.4.2. K-Nearest Neighbors

K-Nearest Neighbors (K-NN) is a non-parametric lazy learning classifier which measures the distance (i.e., via Euclidean, Manhattan methods) between the test instance and every other instance in the training dataset [49]. Then it determines the *K* instances that are closest to the test instance which are finally categorized into the class that most of its K neighbors stem from.

#### 3.4.3. Logistic Regression

Logistic Regression (LR) [50] is a supervised classifier for binary and multinomial tasks. It uses a logistic or a sigmoid function to model the dependent output variable. The model output is dichotomous in nature, i.e., with two possible classes, in which *p* captures the probability of an instance to belong in the HyperChol class; thus, 1−p is the probability of an instance belonging to the Non-HyperChol class. The relationship of log-odds with base *b* and model parameters $\beta_{i}$ is written as:

(2) $$log_{b}\left(\frac{p}{1-p}\right)=\beta_{0}+\beta_{1}y_{i1}+\ldots +\beta_{n}y_{in}$$

#### 3.4.4. Rotation Forest

The Rotation Forest (RotF) [51] applies a rotation transformation matrix to the training before the training of each decision tree to increase the diversity of individual decision trees. A feature reduction technique creates a new feature set for every classifier in the ensemble. It randomly splits the feature set into subsets and applies principal component analysis (PCA) to every created subset separately. Then, a new feature set is acquired by combining principle components of each subset. In this study, the base classifier for the RotF is a J48 [52] decision tree.

#### 3.4.5. Artificial Neural Network

Multilayer Perceptron (MLP) is the simplest fully connected feed-forward Neural Network. It consists of input and output layers and at least one hidden layer. Its neurons are trained by employing back-propagation learning which allows for classification into multiple labels. The MLP is able to learn non-linear models and execute online learning. Finally, it can use any arbitrary activation function [53].

#### 3.4.6. Support Vector Machine

Support Vector Machine (SVM) [54] finds the hyperplane that can optimally separate instances into two classes. The most characteristic kernel functions are linear, polynomial, radial basis and quadratic. An instance $x_{i}$ can be optimally classified based on function:

(3) $$\begin{array}{c} f\left(y^{{}^{\prime }}\right)=Sgn\left[\sum_{i=1}^{M}\alpha_{i}c_{i}K\left(y_{i},y^{{}^{\prime }}\right)+b\right]\\ 0\leq \alpha_{i}\leq C,\;\sum \alpha_{i}c_{i}=0,\;\alpha_{i}\geq 0,i=1,2,\cdots ,M \end{array}$$

where *M* is the size of training instances, $y_{i},c_{i}$ are the training instance feature vector and its class label, respectively, *b* is a bias, $c_{i}\in \left\{1,\;-1\right\}$, $K(y_{i},y^{{}^{\prime }})$ is the kernel function which corresponds the input vectors into an expanded feature space and $f\left(y^{{}^{\prime }}\right)\in \left\{-1,+1\right\}$ is the kernelized binary classifier’s predicted class for the unlabeled instance $y^{{}^{\prime }}$.

#### 3.4.7. Decision Tree

From the available Decision Trees, we considered the Reduced Error Pruning Tree (RepTree) [55]. It is a simple and fast decision learner which builds a decision/regression tree using information gain as an impurity measure and prunes it using reduced-error pruning. RepTree is even more accurate when dealing with a large volume of data.

#### 3.4.8. Logistic Model Tree

A Logistic Model Tree (LMT) [56] consists of a standard decision tree structure with logistic regression functions $f\left(y_{i}\right)=\beta_{0}+\sum_{j=1}^{n}\left(\beta_{i}y_{ij}\right)$ at the leaves. LMTree constructs the tree growing process using the LogitBoost algorithm and the tree pruning is performed using Classification And Regression Tree (CART).

#### 3.4.9. Random Forest

Random Forest (RF) is a bootstrapping technique based on a decision tree with high-performance outcomes, in both regression and classification tasks. It considers the Information Gain or Gini index to find the optimal subset of features, trains multiple decision trees and then classifies an instance by applying majority voting on the results of multiple Decision Trees [57].

#### 3.4.10. Ensemble Learning

**Figure 7:**
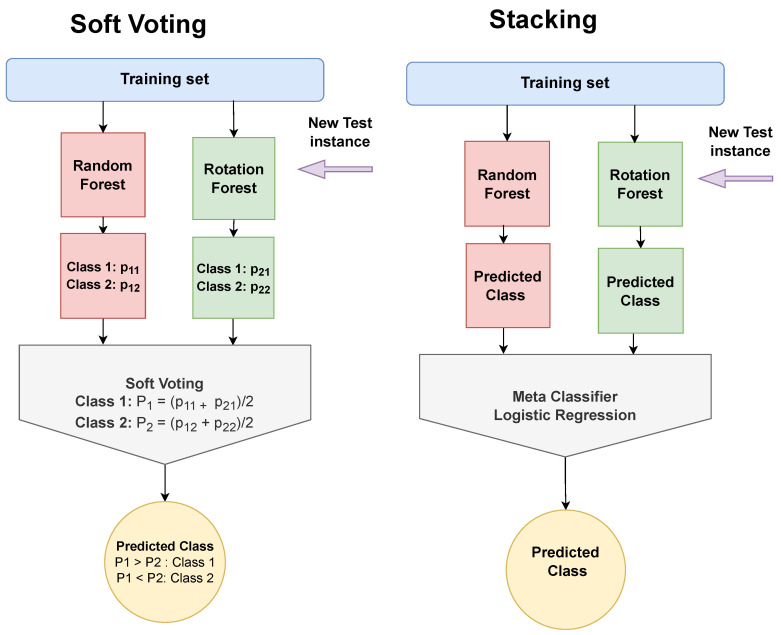
Ensemble Learners: Soft Voting and Stacking.

Ensemble Learning is a machine learning method that combines the outcomes of several single classifiers called base models. Voting and Stacking are two common approaches which are utilized to acquire more accurate predictions than the single models’. Concerning Voting, there are two types, Soft and Hard. Soft Voting, which is exploited in this study, averages the corresponding probabilities of the considered base classifiers and assigns a test instance to the class with the highest probability [58]. On the other hand, Stacking uses the predicted class labels of the base models as input features to train a meta-classifier which undertakes to find the class label [59]. Figure 7 illustrates the two schemes as they will be evaluated in the experiments.

### 3.5. Evaluation Metrics

To assess the ML models’ performance, accuracy, precision, recall, F-measure, and AUC metrics were considered [60].

Precision (or positive predicted value) shows the ratio of positive instances in relation to true and false positive instances. We also considered recall which captures the true positive rate or a model’s sensitivity to identify the participants who actually had HyperChol and correctly considered as positive, concerning all positive participants. Precision is a measure of quality, while recall is a measure of quantity. F-measure is the harmonic mean of precision and recall and allows the evaluation of a model using a single score. Moreover, we computed accuracy, which shows a model’s ability to correctly identify both HyperChol (positive) and Non-HyperChol (negative) instances. Taking into account the confusion matrix, TP, TN, FP and FN denote the number of true positive, true negative, false positive and false negative instances. Based on these quantities, the performance metrics are written as follows:

(4) $$\begin{array}{ccc} \;\;\;\;\;Precision=\frac{TP}{TP+FP}, & \; & \;\;\;\;\;Recall=\frac{TP}{TP+FN}, \end{array}$$

(5) $$\begin{array}{ccc} F-Measure=2\times \frac{Precision\cdot Recall}{Precision+Recall}, & \; & Accuracy=\frac{TN+TP}{TN+TP+FN+FP} \end{array}$$

Another important metric which will be taken into consideration for the models’ evaluation is area under curve (AUC) which takes values in the range [0,1]. AUC is a measure of separability. The ML models’ performance in distinguishing HyperChol from Non-HyperChol instances is captured by AUC. If AUC attains one, it means that the models have the perfect discrimination ability of the two classes distributions.

## 4. Results and Discussion

### 4.1. Experiments Setup

In this section, the ML models performance is evaluated in the WEKA 3.8.6 environment [61]. WEKA is a free JAVA-based data mining tool created and distributed under the GNU General Public License. It provides a library of various models for data preprocessing, classification, clustering, forecasting, visualization, etc. The computing system in which the experiments were conducted has the following characteristics: Intel(R) Core(TM) i7-9750H CPU @ 2.60 GHz 2.59 GHz 16 GB Memory, Windows 10 Home, 64-bit Operating System, x64-based processor. For our experiments, 10-fold cross-validation was applied to measure the models’ efficiency in the balanced dataset of 388 instances. In Table 3, the settings of the considered ML models are shown.

### 4.2. Evaluation

To fully evaluate the effectiveness of models, we should examine both precision and recall since the improvement of precision typically reduces recall and vice versa. However, if the classes’ distribution is uniform, these metrics may achieve the same outcomes.

In Table 4, we show the performance of various models, which were obtained after the application of data cleaning and class balancing. Selecting the two best-performing single classifiers, we combined them under two schemes, Soft Voting and Stacking. All models’ accuracy is higher than 86% except for the 3-NN model, which still achieved an acceptable accuracy of 70.62%. In addition, precision, AUC, recall and F-measure demonstrate the same promising outcomes as the accuracy.

Soft Voting (SoV) is the model with consistently high efficiency in all metrics. Focusing on AUC, which aggregates the classification performance of a model, the SoV model is able to distinguish between HyperChol class and Non-HyperChol class with a chance of 94.5%. In addition, we see that the combination of rotation with the random forest improved the performance of individual models and especially achieved a higher upgrade in the case of RF than in the RotF. Stacking is the second model with excellent separation performance, although its AUC is 8%, 6% and 2% lower than the ones of SoV, RF and RotF, respectively. SoV’s performance superiority lies in the fact that the base-models have been configured to predict probabilities instead of class labels. In the case of Stacking, class labels may add higher uncertainty to the predictions which are provided in the meta-model to learn how to find the best combination of them.

In Table 5, we capture recall and accuracy metrics comparing only single classifiers before [32] and after data cleaning and further restricting features number. A significant performance improvement is observed revealing the role of data quality, class balancing and dimensionality in the classifiers’ performance.

In addition, we observe that our proposed models are superior to the work [32] in terms of recall and accuracy of at least 10%. The only case where we can see similar performance is in the recall of the 5-NN. It should be mentioned that the 3-NN (Table 4) showed better accuracy and recall than the 5-NN with a percentage gap of about 3%.

In conclusion, the performance of Stacking outperforms the prediction performance of each individual model and is closer to the best single model RotF. Soft Voting, which is a probabilistic scheme, achieves higher performance than Stacking since in the meta-level of the latter, the predicted classes are combined using a logistic regression classifier to achieve an output that is a simple linear combination of the predictions of the sub-models. In either case, ensemble schemes indicated promising efficiency compared to the rest of the classifiers.

## 5. Conclusions

In this study, we exploited supervised learning to develop models for the identification of individuals at risk for hypercholesterolemia manifestation based on several risk factors. Healthcare professionals and clinical experts can benefit from such models to prevent the severe consequences of hypercholesterolemia, such as cardiovascular disease. Data exploration through risk factors analysis can help identify associations among the features and HyperChol. A critical aspect of ML models is that they allow medical experts to regularly reassess the associated risk and give proper guidelines and interventions for its management and treatment or prevent its occurrence.

Performance analysis revealed that data preprocessing is an important step for the design of efficient and accurate models for hypercholesterolemia occurrence. The experimental results showed that Soft Voting having as base classifiers the Random and Rotation Forest prevailed with an AUC of 94.5%, precision of 92%, recall of 91.8%, F-measure of 91.7% and an accuracy equal to 91.75%. Hence, it constitutes a candidate HyperChol risk prediction model.

The future purpose of this study is to extend the ML framework via the employment of deep learning methods by applying the Long Short-Term-Memory (LSTM) algorithm and Convolutional Neural Networks (CNN) in the same data comparing the results in terms of accuracy.

## Figures and Tables

**Table 1:** Statistical Description of the Numerical Features in the Balanced Dataset.

	Min	Max	Mean ± stdv
**Age**	50	85	66.4 ± 9.5
**BMI**	18.3	53.1	28.61 ± 5.02
**Waist**	70	148.6	101.76 ± 13.18
**SysBP**	90	201	136.6 ± 20.5
**DiasBP**	13	108	70.27 ± 12.22
**HDL**	19	114	50.94 ± 16.42
**LDL**	51	328	157.6 ± 40.1
**TotalChol**	75	360	208.49 ± 39.69

**Table 2:** Features’ order of importance in the balanced data.

Feature	Pearson Rank	Feature	Gain Ratio	Feature	InfoGain Ratio	Feature	Random Forest (AUPRC)
TotChol	0.6777	TotChol	0.3061	TotChol	0.5633	TotChol	0.3790
LDL	0.6152	LDL	0.2171	LDL	0.3963	LDL	0.3165
HDL	0.1366	DiasBP	0.1142	DiasBP	0.0283	DiasBP	0.0788
DiasBP	0.1148	Gender	0.0085	Gender	0.0085	Age	0.0512
BMI	0.1106	Alcohol Consumption	0.0079	Alcohol Consumption	0.0079	BMI	0.0262
Gender	0.1038	Hypertension	0.0034	Physical Activity	0.0043	Alcohol Consumption	0.0242
Alcohol Consumption	0.1042	Physical Activity	0.0029	Hypertension	0.0034	HDL	0.0182
Age	0.0711	Diabetes	0.0027	Diabetes	0.0019	SysBP	0.0154
Hypertension	0.0681	SysBP	0	SysBP	0	Waist	0.0151
Physical Activity	0.0586	HDL	0	HDL	0	Gender	0.0145
Diabetes	0.0520	BMI	0	BMI	0	Hypertension	0.0124
SysBP	0.0502	Waist	0	Waist	0	Diabetes	0.0000
Waist	0.0192	Age	0	Age	0	Physical Activity	−0.0021

**Table 3:** Machine Learning Models’ Settings.

Model	Parameters
NB	useKernerEstimator = false
LR	ridge = $10^{-8}$, useConjugateGradientDescent = false
LMT	LR modesl at leaves errorOnProbabilities = false, fastRegression = false, numInstances = 15, useAIC = false
DT	noPruning: false, MinVarianceProp = 0.001 numfolds = 3
RotF (using J48)	confidence_factor: 0.25, unpruned: false minimum_instances per_leaf_node default binary split: false
RF	max_depth = 0, numIterations = 100 numFeatures = 0
ANN	hidden layers: ‘a’, learning rate: 0.3 momentum factor 0.2, training time 500
SVM	kernel type: linear
K-NN	K = 3, 5 Search Algorithm: LinearNNSearch with Euclidean
Stacking	Base Models:RF, RotF Meta-model:LR
Soft Voting	Base Models:RF, RotF Average Probabilities

**Table 4:** Performance Evaluation of ML Models.

	Accuracy	Precision	Recall	F-Measure	AUC
**NB**	87.37%	0.877	0.874	0.873	0.931
**SVM**	88.40%	0.884	0.884	0.884	0.884
**LR**	87.63%	0.876	0.876	0.876	0.927
**ANN**	82.73%	0.828	0.827	0.827	0.912
**3-NN**	70.62%	0.707	0.706	0.706	0.758
**RotF**	90.98%	0.911	0.910	0.910	0.939
**LMT**	86.85%	0.869	0.869	0.869	0.928
**RF**	89.69%	0.900	0.897	0.897	0.943
**DT**	88.92%	0.892	0.889	0.889	0.902
**Stacking**	91.24%	0.915	0.912	0.912	0.937
**Soft Voting**	91.75%	0.920	0.918	0.917	0.945

**Table 5:** Performance Comparison of ML Models.

	Recall		Accuracy	
	Proposed models	[32]	Proposed models	[32]
**NB**	87.40%	68.90%	87.37%	62.69%
**SVM**	88.40%	72.70%	88.40%	59.51%
**ANN**	82.70%	66.70%	82.73%	61.42%
**5-NN**	67.30%	67.70%	67.27%	56.56%
**RotF**	91%	69.60%	90.98%	61.86%
**DT**	88.90%	72.20%	88.92%	61.39%
**LMT**	86.90%	73.50%	86.85%	62.99%
**RF**	89.70%	68.80%	89.69%	61.36%
